# 4-[(2*E*)-2-(4-Chloro­benzyl­idene)hydrazinyl­idene]-1-methyl-1,4-dihydro­pyridine monohydrate

**DOI:** 10.1107/S1600536810015709

**Published:** 2010-05-12

**Authors:** Abdullah Aydın, Mehmet Akkurt, Vildan Alptüzün, Orhan Büyükgüngör, Ulrike Holzgrabe, Krzysztof Radacki

**Affiliations:** a Department of Science Education, Faculty of Education, Kastamonu University, 37200 Kastamonu, Turkey; b Department of Physics, Faculty of Arts and Sciences, Erciyes University, 38039 Kayseri, Turkey; c Department of Pharmaceutical Chemistry, Faculty of Pharmacy, Ege University, 35100 Ízmir, Turkey; d Department of Physics, Faculty of Arts and Sciences, Ondokuz Mayıs University, 55139 Samsun, Turkey; e Institute of Pharmacy and Food Chemistry, University of Würzburg, Am Hubland 97074 Würzburg, Germany; f Institute of Inorganic Chemistry, University of Würzburg, Am Hubland 97074 Würzburg, Germany

## Abstract

In the title compound, C_13_H_12_ClN_3_·H_2_O, the organic mol­ecule is almost planar, with a dihedral angle of 3.22 (10)° between the benzene and pyridine rings. The crystal structure is stabilized by O—H⋯N and C—H⋯O hydrogen bonding and π–π stacking inter­actions [centroid–centroid distances = 3.630 (1) and 3.701 (1) Å].

## Related literature

For the synthesis and pharmacological activity of (benzyl­idene-hydrazono)-1,4-dihydro­pyridine derivatives, see: Douglas *et al.* (1977 ▶); Alptüzün *et al.* (2010 ▶); Savini *et al.* (2002 ▶); Pandey *et al.* (2002 ▶); Salgın-Gökşen *et al.* (2007 ▶); Silva *et al.* (2004 ▶); Vicini *et al.* (2009 ▶). For bond-length data, see: Allen *et al.* (1987 ▶); Diao *et al.* (2008 ▶); Odabaşoğlu *et al.* (2003 ▶). For quantum-chemical calculations, see: Pople & Beveridge (1970 ▶).
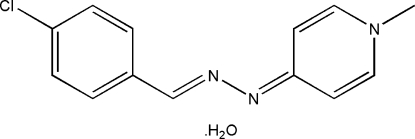


## Experimental

### 

#### Crystal data

C_13_H_12_ClN_3_·H_2_O
*M* *_r_* = 263.72
Monoclinic, 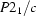

*a* = 5.8492 (4) Å
*b* = 20.3101 (10) Å
*c* = 12.2035 (7) Å
β = 113.855 (4)°
*V* = 1325.90 (14) Å^3^
*Z* = 4
Mo *K*α radiation
μ = 0.28 mm^−1^
*T* = 296 K
0.60 × 0.30 × 0.04 mm

#### Data collection

Stoe IPDS 2 diffractometer
Absorption correction: integration (*X-RED32*; Stoe & Cie, 2002 ▶) *T*_min_ = 0.905, *T*_max_ = 0.989
14028 measured reflections
2759 independent reflections
1746 reflections with *I* > 2σ(*I*)
*R*_int_ = 0.064

#### Refinement

*R*[*F* ^2^ > 2σ(*F* ^2^)] = 0.045
*wR*(*F* ^2^) = 0.095
*S* = 0.95
2759 reflections
170 parameters
H atoms treated by a mixture of independent and constrained refinement
Δρ_max_ = 0.16 e Å^−3^
Δρ_min_ = −0.18 e Å^−3^

### 

Data collection: *X-AREA* (Stoe & Cie, 2002 ▶); cell refinement: *X-AREA*; data reduction: *X-RED32* (Stoe & Cie, 2002 ▶); program(s) used to solve structure: *SIR97* (Altomare *et al.*, 1999 ▶); program(s) used to refine structure: *SHELXL97* (Sheldrick, 2008 ▶); molecular graphics: *ORTEP-3 for Windows* (Farrugia, 1997 ▶); software used to prepare material for publication: *WinGX* (Farrugia, 1999 ▶).

## Supplementary Material

Crystal structure: contains datablocks global, I. DOI: 10.1107/S1600536810015709/sj2782sup1.cif

Structure factors: contains datablocks I. DOI: 10.1107/S1600536810015709/sj2782Isup2.hkl

Additional supplementary materials:  crystallographic information; 3D view; checkCIF report

## Figures and Tables

**Table 1:** Hydrogen-bond geometry (Å, °)

*D*—H⋯*A*	*D*—H	H⋯*A*	*D*⋯*A*	*D*—H⋯*A*
O1—H1*A*⋯N1	0.84 (3)	2.26 (3)	3.089 (3)	173 (3)
O1—H1*B*⋯N2^i^	0.91 (4)	1.95 (4)	2.859 (3)	174 (2)
C3—H3⋯O1^ii^	0.93	2.58	3.421 (3)	150
C11—H11⋯O1^iii^	0.93	2.49	3.378 (3)	159

Symmetry codes: (i) 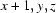; (ii) 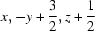; (iii) 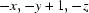.

**Fig. 1.**
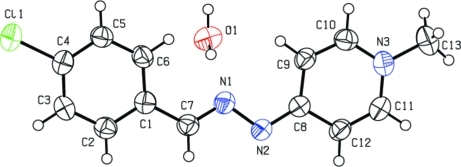
An ORTEP View of the title molecule with the atom numbering scheme. Displacement ellipsoids for non-H atoms are drawn at the 50% probability level.

**Fig. 2.**
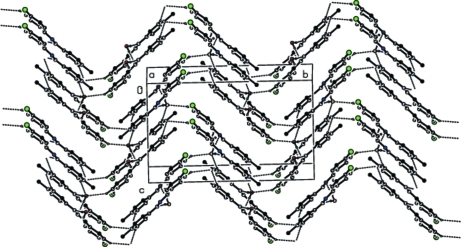
The packing and hydrogen bonding interactions of (I) down the a-axis. H atoms not participating in hydrogen bonding have been omitted for clarity.

**Fig. 3.**
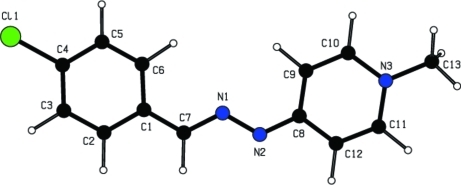
The spatial view of the title molecule (I), calculated by the CNDO aproximation.

## supplementary crystallographic information

### Comment

Hydrazones, a special group of compounds in the class of the schiff bases, are known to show significant biological activities including antimicrobial, antitubercular, anticancer, analgesic, anti-inflammatory, antiplatelet and antiviral activities (Savini *et al.*, 2002; Pandey *et al.*, 2002; Salgın-Gökşen *et al.*, 2007; Silva *et al.*, 2004; Vicini *et al.*, 2009). In addition, (benzylidene-hydrazono)-1,4-dihydropyridine derivatives have anticoccidial activity (Douglas *et al.*, 1977) and also display anti-Alzheimer's activity by inhibiting ABeta fibril formation and acetylcholinesterase (Alptüzün *et al.*, 2010).

The title molecule (I), Fig. 1, crystallized as a monohydrate in the monoclinic space group *P*2_1_/c. All bond lengths are as expected (Allen *et al.*, 1987). The Cl1—C4, and N1—N2 bond lengths are 1.742 (2) Å, and 1.388 (2) Å, respectively. The Cl1—C4—C5 and N2—N1—C7 bond angles are 119.88 (18) ° and 113.95 (19) °, respectively. The bond lengths and the bond angles of (I) are comparable to those observed in related structures (Diao *et al.*, 2008; Odabaşoğlu *et al.*, 2003).

The main molecule is almost planar, except the methyl H atoms, forming a dihedral angle of 3.22 (10)° between the benzene (C1–C6) and dihydropyridine (N3/C8–C12) rings.

The crystal structure is stabilized by O—H···N and C—H···O hydrogen bonding (Table 1, Fig. 2) and π-π stacking interactions [Cg1···Cg1(-x, 1-y, -z) = 3.630 (1) Å and Cg1···Cg2(1-x, 1-y, 1-z) = 3.701 (1) Å, Cg1 and Cg2 are the centroids of the pyridine and benzene rings, respectively].

We have also carried out the quantum mechanical calculations using the *CNDO* (Pople *et al.*, 1970) approximation. The spatial view of the single molecule considered in a vacuum, is shown in Fig.3. According to the theoretical *CNDO* and experimental X-rays results, the values of the geometric parameters of (I) are closely comparable within the observed experimental errors.  The calculated dipole moment of (I) is about 11.481 Debye. The *HOMO* and *LUMO* energy levels are -8.3484 and 1.3565 eV, respectively.

### Experimental

4-Hydrazinylpyridine (1.09 g, 0.01 mol) and 4-chlorobenzaldehyde (1.41 g, 0.01 mol) were stirred in ethanol (30 ml) at room temperature for 5-10 h. The precipitate was filtered and washed with cool ethanol and crystallized from ethanol. A mixture of 4-[(4-Chlorobenzylidene)hydrazinyl] pyridine (0.232 g, 0.001 mol) and methyl iodide (0.141 g, 0.002 mol) was refluxed in ethanol (20 ml) for 20 h. The mixture was cooled to room temperature and the resulting precipitate was filtered and washed with cool ethanol. The crude products were crystallized from ethanol to give the compound 4-(2-(4-Chlorobenzylidenehydrazinyl)-1-methylpyridinium iodide. This product (0.374 g, 0.001 mol) was partitioned between CH_2_Cl_2_ (50 ml) and 2 M NaOH (50 ml).  The organic layer was evaporated to dryness and the residue recrystallized from ethanol-water.

Yield 88%, yellow needles, mp 407-409 K (lit. (Douglas *et al.* , 1977) 403-405 K). IR (KBr) *v*_max_ 1654, 1517, 1492, 1203, 824 cm^-1^. ^1^H-NMR (DMSO-d_6_): δ ppm 3.29 (3*H*, s, N—CH_3_), 6.12 (1*H*, dd, J=2.4/8.0 Hz, H-3 or H-5), 6.98 (1*H*, dd, J=2.0/7.8 Hz, H-3 or H-5), 7.23 (2*H*, td, J=7.2/2.0 Hz, H-2, H-6 ), 7.39 (2*H*, d, J=8.4 Hz, H-2', H-6'), 7.69 (2*H*, d, J=8.4 Hz, H-3', H-5'), 8.16 (1*H*, s, N=CH). 13 C NMR (CH_3_OH- d4): δ ppm 43.18 (q), 107.78 (d), 112.11 (d), 129.35 (d), 129.79 (d), 135.59 (s), 136.58 (s), 140.15 (d), 140.75 (d), 148.84 (d), 162.25 (s). EI—MS m/z (% relative intensity): 247 (M+2, 14), 246 (M+1, 28), 245 (M+, 43), 181 (25), 93 (100), 92 (24), 66 (30), 42 (18). C_13_H_12_N_3_Cl.H_2_O. C, H, N combustion analysis: Calc. (%) C 59.21, H 5.36, N 15.93; found (%) C 59.45, H 5.33, N 15.72.

### Refinement

The H atoms of the water molecule were found from a difference Fourier map and their isotropic thermal parameters were refined by using a riding model with U_iso_(H) = 1.5U_eq_(O). Their positional parameters are refined freely [d(O–H) = 0.84 (3) and 0.91 (4) Å]. The remaining H atoms were positioned geometrically and refined using a riding model with C—H = 0.93 and 0.96 Å, and *U*_iso_(H) = 1.2 or 1.5*U*_eq_(C).

### Crystal data

**Table:**

C_13_H_12_ClN_3_·H_2_O	*F*(000) = 552
*M**_r_* = 263.72	*D*_x_ = 1.321 Mg m^−^^3^
Monoclinic, *P*2_1_/*c*	Mo *K*α radiation, λ = 0.71073 Å
Hall symbol: -P 2ybc	Cell parameters from 11448 reflections
*a* = 5.8492 (4) Å	θ = 1.8–27.3°
*b* = 20.3101 (10) Å	µ = 0.28 mm^−^^1^
*c* = 12.2035 (7) Å	*T* = 296 K
β = 113.855 (4)°	Needle, yellow
*V* = 1325.90 (14) Å^3^	0.60 × 0.30 × 0.04 mm
*Z* = 4	

### Data collection

**Table:**

Stoe IPDS 2 diffractometer	2759 independent reflections
Radiation source: sealed X-ray tube, 12 x 0.4 mm long-fine focus	1746 reflections with *I* > 2σ(*I*)
plane graphite	*R*_int_ = 0.064
Detector resolution: 6.67 pixels mm^-1^	θ_max_ = 26.5°, θ_min_ = 2.0°
ω scans	*h* = −7→7
Absorption correction: integration (*X-RED32*; Stoe & Cie, 2002)	*k* = −25→25
*T*_min_ = 0.905, *T*_max_ = 0.989	*l* = −15→15
14028 measured reflections	

### Refinement

**Table:**

Refinement on *F*^2^	Primary atom site location: structure-invariant direct methods
Least-squares matrix: full	Secondary atom site location: difference Fourier map
*R*[*F*^2^ > 2σ(*F*^2^)] = 0.045	Hydrogen site location: inferred from neighbouring sites
*wR*(*F*^2^) = 0.095	H atoms treated by a mixture of independent and constrained refinement
*S* = 0.95	*w* = 1/[σ^2^(*F*_o_^2^) + (0.0426*P*)^2^] where *P* = (*F*_o_^2^ + 2*F*_c_^2^)/3
2759 reflections	(Δ/σ)_max_ < 0.001
170 parameters	Δρ_max_ = 0.16 e Å^−^^3^
0 restraints	Δρ_min_ = −0.18 e Å^−^^3^

### Special details

**Table:**

Geometry. Bond distances, angles etc. have been calculated using the rounded fractional coordinates. All su's are estimated from the variances of the (full) variance-covariance matrix. The cell esds are taken into account in the estimation of distances, angles and torsion angles
Refinement. Refinement on *F*^2^ for ALL reflections except those flagged by the user for potential systematic errors. Weighted *R*-factors wR and all goodnesses of fit *S* are based on *F*^2^, conventional *R*-factors *R* are based on *F*, with *F* set to zero for negative *F*^2^. The observed criterion of *F*^2^ > σ(*F*^2^) is used only for calculating -*R*-factor-obs etc. and is not relevant to the choice of reflections for refinement. *R*-factors based on *F*^2^ are statistically about twice as large as those based on *F*, and *R*-factors based on ALL data will be even larger.

### Fractional atomic coordinates and isotropic or equivalent isotropic displacement parameters (Å^2^)

**Table:**

	*x*	*y*	*z*	*U*_iso_*/*U*_eq_	
Cl1	1.22894 (12)	0.72508 (3)	0.80784 (5)	0.0626 (2)	
N1	0.3123 (3)	0.56701 (10)	0.35465 (13)	0.0453 (6)	
N2	0.0774 (3)	0.54841 (9)	0.27204 (13)	0.0441 (6)	
N3	0.0599 (4)	0.39257 (9)	0.05147 (14)	0.0478 (6)	
C1	0.5371 (4)	0.63831 (11)	0.52012 (15)	0.0414 (7)	
C2	0.5222 (4)	0.69371 (12)	0.58483 (16)	0.0452 (7)	
C3	0.7336 (4)	0.72021 (11)	0.67306 (16)	0.0466 (7)	
C4	0.9614 (4)	0.69105 (12)	0.69752 (16)	0.0449 (7)	
C5	0.9807 (4)	0.63606 (12)	0.63540 (16)	0.0460 (8)	
C6	0.7697 (4)	0.60996 (12)	0.54740 (16)	0.0460 (7)	
C7	0.3079 (4)	0.61193 (11)	0.42743 (16)	0.0435 (7)	
C8	0.0859 (4)	0.49832 (11)	0.20400 (15)	0.0396 (7)	
C9	0.3019 (4)	0.46263 (11)	0.21152 (16)	0.0437 (7)	
C10	0.2819 (4)	0.41223 (12)	0.13694 (18)	0.0480 (7)	
C11	−0.1504 (4)	0.42521 (12)	0.04046 (17)	0.0473 (7)	
C12	−0.1430 (4)	0.47578 (12)	0.11261 (16)	0.0449 (7)	
C13	0.0497 (5)	0.33651 (13)	−0.0272 (2)	0.0668 (10)	
O1	0.6224 (4)	0.61903 (10)	0.21954 (15)	0.0611 (7)	
H2	0.36750	0.71300	0.56810	0.0540*	
H3	0.72240	0.75730	0.71540	0.0560*	
H5	1.13590	0.61680	0.65300	0.0550*	
H6	0.78280	0.57290	0.50560	0.0550*	
H7	0.15450	0.62820	0.42110	0.0520*	
H9	0.45830	0.47440	0.26870	0.0520*	
H10	0.42620	0.38990	0.14420	0.0580*	
H11	−0.30330	0.41230	−0.01840	0.0570*	
H12	−0.29140	0.49680	0.10280	0.0540*	
H13A	0.18000	0.34090	−0.05550	0.1000*	
H13B	−0.10970	0.33600	−0.09420	0.1000*	
H13C	0.07200	0.29620	0.01700	0.1000*	
H1A	0.551 (6)	0.6046 (17)	0.262 (2)	0.0920*	
H1B	0.768 (6)	0.5959 (17)	0.242 (2)	0.0920*	

### Atomic displacement parameters (Å^2^)

**Table:**

	*U*^11^	*U*^22^	*U*^33^	*U*^12^	*U*^13^	*U*^23^
Cl1	0.0551 (4)	0.0646 (4)	0.0574 (3)	−0.0114 (3)	0.0116 (2)	−0.0153 (3)
N1	0.0411 (10)	0.0499 (12)	0.0415 (8)	−0.0024 (9)	0.0133 (7)	−0.0039 (8)
N2	0.0365 (10)	0.0484 (12)	0.0446 (8)	−0.0022 (9)	0.0135 (7)	−0.0053 (8)
N3	0.0558 (12)	0.0433 (12)	0.0455 (9)	−0.0060 (10)	0.0216 (8)	−0.0054 (8)
C1	0.0461 (13)	0.0438 (13)	0.0351 (9)	−0.0015 (11)	0.0173 (9)	0.0023 (8)
C2	0.0453 (13)	0.0457 (14)	0.0446 (10)	0.0042 (11)	0.0182 (9)	0.0017 (9)
C3	0.0577 (14)	0.0395 (13)	0.0434 (10)	0.0004 (12)	0.0212 (10)	−0.0043 (9)
C4	0.0472 (13)	0.0469 (14)	0.0387 (10)	−0.0069 (11)	0.0154 (9)	0.0011 (9)
C5	0.0431 (13)	0.0481 (15)	0.0456 (11)	0.0046 (11)	0.0167 (10)	−0.0020 (9)
C6	0.0485 (14)	0.0474 (14)	0.0406 (10)	0.0035 (11)	0.0164 (9)	−0.0065 (9)
C7	0.0422 (12)	0.0473 (14)	0.0429 (10)	0.0003 (11)	0.0191 (9)	−0.0014 (9)
C8	0.0393 (12)	0.0407 (13)	0.0388 (9)	−0.0004 (10)	0.0158 (8)	0.0028 (9)
C9	0.0374 (12)	0.0458 (14)	0.0428 (10)	−0.0013 (10)	0.0110 (9)	0.0001 (9)
C10	0.0456 (13)	0.0460 (14)	0.0545 (11)	0.0041 (11)	0.0223 (10)	0.0025 (10)
C11	0.0424 (13)	0.0503 (15)	0.0437 (10)	−0.0070 (12)	0.0118 (9)	−0.0004 (10)
C12	0.0357 (12)	0.0511 (15)	0.0439 (10)	−0.0021 (11)	0.0120 (9)	−0.0015 (9)
C13	0.084 (2)	0.0570 (17)	0.0615 (14)	−0.0065 (15)	0.0316 (14)	−0.0174 (12)
O1	0.0514 (11)	0.0661 (13)	0.0668 (10)	0.0032 (9)	0.0250 (8)	0.0088 (8)

### Geometric parameters (Å, °)

**Table:**

Cl1—C4	1.742 (2)	C8—C9	1.427 (3)
O1—H1B	0.91 (4)	C8—C12	1.428 (3)
O1—H1A	0.84 (3)	C9—C10	1.343 (3)
N1—N2	1.388 (2)	C11—C12	1.342 (3)
N1—C7	1.281 (3)	C2—H2	0.9300
N2—C8	1.327 (3)	C3—H3	0.9300
N3—C10	1.356 (3)	C5—H5	0.9300
N3—C11	1.355 (3)	C6—H6	0.9300
N3—C13	1.475 (3)	C7—H7	0.9300
C1—C2	1.398 (3)	C9—H9	0.9300
C1—C7	1.462 (3)	C10—H10	0.9300
C1—C6	1.388 (3)	C11—H11	0.9300
C2—C3	1.379 (3)	C12—H12	0.9300
C3—C4	1.376 (3)	C13—H13C	0.9600
C4—C5	1.380 (3)	C13—H13A	0.9600
C5—C6	1.373 (3)	C13—H13B	0.9600
			
Cl1···C2^i^	3.521 (2)	C11···C8^iv^	3.516 (3)
Cl1···C7^i^	3.572 (2)	C11···O1^iv^	3.378 (3)
Cl1···H10^ii^	2.9800	C13···C4^xi^	3.598 (3)
O1···N2^iii^	2.859 (3)	C13···C3^xi^	3.490 (4)
O1···C11^iv^	3.378 (3)	C3···H13A^x^	2.9800
O1···N1	3.089 (3)	C7···H1B^vii^	3.07 (3)
O1···H13B^iv^	2.9100	C7···H1A	2.91 (3)
O1···H3^v^	2.5800	C8···H1B^vii^	2.88 (4)
O1···H11^iv^	2.4900	C12···H1B^vii^	3.06 (3)
O1···H13A^vi^	2.8100	H1A···N1	2.26 (3)
N1···O1	3.089 (3)	H1A···C7	2.91 (3)
N2···O1^vii^	2.859 (3)	H1B···H12^iii^	2.5700
N1···H1A	2.26 (3)	H1B···N2^iii^	1.95 (4)
N1···H6	2.6200	H1B···C7^iii^	3.07 (3)
N1···H9	2.4700	H1B···C8^iii^	2.88 (4)
N2···H1B^vii^	1.95 (4)	H1B···C12^iii^	3.06 (3)
C2···Cl1^viii^	3.521 (2)	H1B···H7^iii^	2.5100
C3···C10^ix^	3.576 (3)	H2···H7	2.4300
C3···C13^x^	3.490 (4)	H3···O1^xii^	2.5800
C4···C13^x^	3.598 (3)	H6···N1	2.6200
C4···C10^ix^	3.582 (3)	H7···H1B^vii^	2.5100
C5···C8^ix^	3.473 (3)	H7···H2	2.4300
C5···C9^ix^	3.569 (3)	H9···N1	2.4700
C6···C9^ix^	3.464 (3)	H10···H13A	2.4800
C6···C8^ix^	3.561 (3)	H10···Cl1^ii^	2.9800
C7···Cl1^viii^	3.572 (2)	H11···H13B	2.3200
C8···C11^iv^	3.516 (3)	H11···O1^iv^	2.4900
C8···C6^ix^	3.561 (3)	H12···H1B^vii^	2.5700
C8···C5^ix^	3.473 (3)	H13A···H10	2.4800
C9···C5^ix^	3.569 (3)	H13A···C3^xi^	2.9800
C9···C6^ix^	3.464 (3)	H13A···O1^vi^	2.8100
C10···C4^ix^	3.582 (3)	H13B···H11	2.3200
C10···C3^ix^	3.576 (3)	H13B···O1^iv^	2.9100
			
H1A—O1—H1B	106 (3)	C1—C2—H2	120.00
N2—N1—C7	113.95 (19)	C3—C2—H2	120.00
N1—N2—C8	112.75 (18)	C4—C3—H3	120.00
C10—N3—C13	120.1 (2)	C2—C3—H3	120.00
C11—N3—C13	121.1 (2)	C4—C5—H5	120.00
C10—N3—C11	118.73 (19)	C6—C5—H5	120.00
C2—C1—C6	118.52 (19)	C5—C6—H6	120.00
C6—C1—C7	122.5 (2)	C1—C6—H6	120.00
C2—C1—C7	119.0 (2)	N1—C7—H7	119.00
C1—C2—C3	121.0 (2)	C1—C7—H7	119.00
C2—C3—C4	119.0 (2)	C10—C9—H9	120.00
Cl1—C4—C5	119.88 (18)	C8—C9—H9	120.00
C3—C4—C5	121.1 (2)	N3—C10—H10	119.00
Cl1—C4—C3	119.03 (17)	C9—C10—H10	119.00
C4—C5—C6	119.7 (2)	C12—C11—H11	119.00
C1—C6—C5	120.7 (2)	N3—C11—H11	119.00
N1—C7—C1	121.9 (2)	C8—C12—H12	119.00
N2—C8—C12	118.3 (2)	C11—C12—H12	119.00
C9—C8—C12	114.43 (19)	N3—C13—H13B	109.00
N2—C8—C9	127.27 (19)	N3—C13—H13C	109.00
C8—C9—C10	120.8 (2)	N3—C13—H13A	109.00
N3—C10—C9	122.7 (2)	H13A—C13—H13C	110.00
N3—C11—C12	121.5 (2)	H13B—C13—H13C	110.00
C8—C12—C11	122.0 (2)	H13A—C13—H13B	109.00
			
C7—N1—N2—C8	−174.99 (18)	C6—C1—C7—N1	−10.1 (3)
N2—N1—C7—C1	−179.43 (18)	C1—C2—C3—C4	−0.4 (3)
N1—N2—C8—C12	−178.00 (18)	C2—C3—C4—C5	0.0 (3)
N1—N2—C8—C9	2.6 (3)	C2—C3—C4—Cl1	179.29 (17)
C13—N3—C11—C12	179.2 (2)	C3—C4—C5—C6	0.2 (3)
C10—N3—C11—C12	−0.6 (3)	Cl1—C4—C5—C6	−179.11 (17)
C11—N3—C10—C9	0.3 (3)	C4—C5—C6—C1	0.1 (3)
C13—N3—C10—C9	−179.5 (2)	N2—C8—C9—C10	179.3 (2)
C2—C1—C6—C5	−0.5 (3)	N2—C8—C12—C11	−179.7 (2)
C7—C1—C2—C3	180.0 (2)	C9—C8—C12—C11	−0.2 (3)
C7—C1—C6—C5	−179.8 (2)	C12—C8—C9—C10	−0.2 (3)
C2—C1—C7—N1	170.6 (2)	C8—C9—C10—N3	0.1 (3)
C6—C1—C2—C3	0.7 (3)	N3—C11—C12—C8	0.5 (3)

Symmetry codes: (i) *x*+1, −*y*+3/2, *z*+1/2; (ii) −*x*+2, −*y*+1, −*z*+1; (iii) *x*+1, *y*, *z*; (iv) −*x*, −*y*+1, −*z*; (v) *x*, −*y*+3/2, *z*−1/2; (vi) −*x*+1, −*y*+1, −*z*; (vii) *x*−1, *y*, *z*; (viii) *x*−1, −*y*+3/2, *z*−1/2; (ix) −*x*+1, −*y*+1, −*z*+1; (x) −*x*+1, *y*+1/2, −*z*+1/2; (xi) −*x*+1, *y*−1/2, −*z*+1/2; (xii) *x*, −*y*+3/2, *z*+1/2.

### Hydrogen-bond geometry (Å, °)

**Table:**

*D*—H···*A*	*D*—H	H···*A*	*D*···*A*	*D*—H···*A*
O1—H1A···N1	0.84 (3)	2.26 (3)	3.089 (3)	173 (3)
O1—H1B···N2^iii^	0.91 (4)	1.95 (4)	2.859 (3)	174 (2)
C3—H3···O1^xii^	0.93	2.58	3.421 (3)	150
C11—H11···O1^iv^	0.93	2.49	3.378 (3)	159

Symmetry codes: (iii) *x*+1, *y*, *z*; (xii) *x*, −*y*+3/2, *z*+1/2; (iv) −*x*, −*y*+1, −*z*.
